# Influence of Scanner Precision and Analysis Software in Quantifying Three-Dimensional Intraoral Changes: Two-Factor Factorial Experimental Design

**DOI:** 10.2196/17150

**Published:** 2020-11-27

**Authors:** Saoirse O'Toole, David Bartlett, Andrew Keeling, John McBride, Eduardo Bernabe, Luuk Crins, Bas Loomans

**Affiliations:** 1 Centre for Clinical, Oral and Translational Sciences King’s College London London United Kingdom; 2 University of Leeds Leeds United Kingdom; 3 University of Southampton Southampton United Kingdom; 4 Radboud University Medical Centre Nijmegen Netherlands

**Keywords:** diagnostic systems, digital imaging/radiology, engineering, imaging, outcomes research, tooth wear

## Abstract

**Background:**

Three-dimensional scans are increasingly used to quantify biological topographical changes and clinical health outcomes. Traditionally, the use of 3D scans has been limited to specialized centers owing to the high cost of the scanning equipment and the necessity for complex analysis software. Technological advances have made cheaper, more accessible methods of data capture and analysis available in the field of dentistry, potentially facilitating a primary care system to quantify disease progression. However, this system has yet to be compared with previous high-precision methods in university hospital settings.

**Objective:**

The aim of this study was to compare a dental primary care method of data capture (intraoral scanner) with a precision hospital-based method (laser profilometer) in addition to comparing open source and commercial software available for data analysis.

**Methods:**

Longitudinal dental wear data from 30 patients were analyzed using a two-factor factorial experimental design. Bimaxillary intraoral digital scans (TrueDefinition, 3M, UK) and conventional silicone impressions, poured in type-4 dental stone, were made at both baseline and follow-up appointments (mean 36 months, SD 10.9). Stone models were scanned using precision laser profilometry (Taicaan, Southampton, UK). Three-dimensional changes in both forms of digital scans of the first molars (n=76) were quantitatively analyzed using the engineering software Geomagic Control (3D Systems, Germany) and freeware WearCompare (Leeds Digital Dentistry, UK). Volume change (mm^3^) was the primary measurement outcome. The maximum point loss (μm) and the average profile loss (μm) were also recorded. Data were paired and skewed, and were therefore compared using Wilcoxon signed-rank tests with Bonferroni correction.

**Results:**

The median (IQR) volume change for Geomagic using profilometry and using the intraoral scan was –0.37 mm^3^ (–3.75-2.30) and +0.51 mm^3^ (–2.17-4.26), respectively (*P*<.001). Using WearCompare, the median (IQR) volume change for profilometry and intraoral scanning was –1.21 mm^3^ (–3.48-0.56) and –0.39 mm^3^ (–3.96-2.76), respectively (*P*=.04). WearCompare detected significantly greater volume loss than Geomagic regardless of scanner type. No differences were observed between groups with respect to the maximum point loss or average profile loss.

**Conclusions:**

As expected, the method of data capture, software used, and measurement metric all significantly influenced the measurement outcome. However, when appropriate analysis was used, the primary care system was able to quantify the degree of change and can be recommended depending on the accuracy needed to diagnose a condition. Lower-resolution scanners may underestimate complex changes when measuring at the micron level.

## Introduction

All clinicians should be able to quantify and assess whether a degenerative health condition is stable or progressing. This is possible in some diseases that have accurate biomarkers but is not always possible for diseases of the soft and hard tissues. Measurement has typically taken the form of recording subjective visual changes; therefore, physical measurements of change are needed.

In dentistry, quantitative measurement of differences between sequential 3D scans of the teeth is typically used to diagnose erosive tooth wear. This is a condition in which excessive acids from the diet and stomach can dissolve the teeth. Due to changes in diet and health, the prevalence of erosive tooth wear has been increasing, now affecting 1 in every 3 adults globally [1]. Quantitative validation of tooth wear has only been possible in university hospitals thus far [2-5]. This validation is achieved by scanning accurate molds of the teeth with laser profilometers to create a precise digital map of the surface with repeatable, calibrated point coordinates. As directly scanning teeth with lab-based profilometers has not been possible, scans of molds of the teeth have been aligned and compared using custom-built or commercial engineering software to quantify changes. Data capture is typically very accurate with this approach, and small process errors have been calculated to be in the range of 15 microns [6,7]. However, the reliance on research laboratory–based scanners and complex engineering analysis software is expensive and unfeasible for use in primary care settings [6-8].

Digital handheld scanners, known as intraoral scanners, take digital maps of the teeth and are increasingly being used in primary care. Intraoral scanners do not generate aerosols and are more amenable to effective cross-infection control compared to conventional impressions that generate aerosols and can harbor pathogenic microorganisms [9]. These advantages are particularly relevant for ensuring complete disinfection during the current COVID-19 pandemic. Intraoral scanners capture data via different methods, ranging from video capture to the use of confocal, triangulation, or active wavefront principles. Rather than relying on accurate calibrated data point collection on an unmoving subject, multiple data points are captured and stitched together with company-specific algorithms. Errors are generated when the scanner fails to collect sufficient data to stitch a digital map of the surface (undersampling) [10,11] or when the process fails, particularly with more than one tooth [12,13]. Furthermore, data stitching algorithms often interpolate or smooth missing or erroneous data; therefore, the data points are estimated, nonuniform, and lack adequate surface detail for changes to be measured at the micron level.

The software currently used by commercial companies to analyze digital maps rely on an iterative closest point (ICP) algorithm to merge the maps to the closest possible alignment, without considering if the proposed alignment solution makes biological sense [14]. We previously demonstrated that this estimation leads to distortions and can result in physiologically impossible outcomes [15,16]. We recently incorporated feature-recognizing elements [16,17] into an ICP algorithm to minimize these errors and created an open-source freeware to be used alongside any 3D scan. Although this method has been validated against previous gold-standard software [18], it has not yet been tested on longitudinal clinical data.

The combination of data collection from primary care and free, user-friendly software for analysis may create new opportunities for monitoring disease. However, the accuracy of measuring change in scans will be influenced by the scanner, software, and their interaction. In this study, we used a factorial design to compare data obtained from profilometric scans of casts and those obtained from direct intraoral scans using two types of registered software: commercial software (Geomagic Control, 3D Systems, Germany) and freeware (WearCompare, Leeds Digital Dentistry, UK). We expected to see differences in the measurements obtained between the scanners but we did not know whether this difference would be clinically significant. The primary null hypothesis was that the dental wear data, specifically the volume change, average profile loss, and maximum point loss, detected by the profilometer will not be different to those obtained with the intraoral scanner. The secondary null hypothesis was that the software used to analyze the data will not influence the volume change, average profile loss, and maximum point loss observed for either scanner.

## Methods

### Participants

Data were collected from a larger clinical longitudinal erosive tooth wear study (Radboud Tooth Wear Project ABR code: NL31371.091.10) [19,20]. Study participants had been referred by general dental practitioners to the Department of Dentistry of Radboud University Medical Center (Nijmegen, the Netherlands) for management of erosive tooth wear. Patients in the monitoring arm who provided additional written consent for their data to be transported to the United Kingdom (ABR codes NL31401.091.10) and to perform additional analyses were included in the study (n=25; mean age 35.8, SD 6.8 years; 20 men and 5 women). A power calculation was performed in GPower vs 3.1 [21] using a two tailed test, demonstrating that to obtain a correlation of 0.4 between the scanners at 95% power with *P*<.05, a sample size of 75 was required.

### Digital and Dental Impressions

The data transported to the United Kingdom included digital intraoral scans obtained using Lava Chairside Oral Scanner (3M, USA) at baseline and 3M True Definition Intraoral Scanner (3M, UK) at follow up, and analog dental impressions taken with silicone (Ivoclar Virtual 380, Ivoclar Vivodent, Liechtenstein, Europe). Impressions were poured in type-3 dental stone (SLR Dental GmbH, Germany) within 24 hours according to the manufacturer’s instructions.

Both the digital and dental impressions were captured by the same trained operator. The point clouds of recognized index teeth (ie, the occlusal surface of the first molars [22,23]) were isolated by the operator (ST) and set aside for evaluation. Each analog study model (n=100) was scanned using a noncontact triangulation laser profilometer (XYRIS 2000TL, Taicaan Technologies, Southampton, UK) in a raster pattern using a step-over of 50 µm with a repeatability error of 2.6 µm [24]. This generated a 3D point cloud dataset for comparison.

### Measurements and Software

Quantitative analysis of the change between sequential scans from the profilometer and intraoral scanner was performed using both the commercial software Geomagic Control 2011 (Geomagic, Morrisville, NC, USA) and the freeware WearCompare (Leeds Digital Dentistry, Leeds, UK). Data points selected by the operator on the buccal and lingual surfaces were chosen as reference areas and used for analysis according to previously published protocols [16]. For Geomagic, a best-fit alignment of 1000 data points on reference surfaces, followed by a refined alignment using 5000 data points, was performed. For the reference alignment, the occlusal surface was deleted from the dataset, leaving the buccal and lingual reference surfaces. The transformation matrix was then applied to the complete displaced dataset to realign it with the same orientation. For WearCompare, an initial global alignment utilizing a feature-based recognition system was performed. The same buccal and lingual reference surfaces were selected for refined ICP alignments, which highlights corresponding reference areas within 25 microns of each other. The occlusal surface was selected to be measured and all measurements were taken perpendicular to the occlusal surface.

Volume change (mm^3^), maximum point loss (μm), and the mean loss over the surface (μm) were analyzed for each surface for both scanners and software types. As a secondary volumetric analysis, any positive values, indicating either gain or error, were set to zero.

### Statistical Analysis

This study utilized a two-factor factorial experimental design comparing two different methods of data capture (profilometer and intraoral scanner) and two different analysis software types with different alignment principles (Geomagic Control and WearCompare). Descriptive statistics of all measurement metrics were calculated, and normality was assessed using the Shapiro-Wilks test and histogram assessment. Since the data were paired and skewed, Wilcoxon signed-rank tests were used to compare outcomes (volume change, maximum point loss, and the average loss over the surface) between groups. Bonferroni correction was applied to compensate for multiple comparisons. The significance level was set at .008 (.05/6) to identify differences between groups. Single-measures intraclass correlation (ICC) analysis was performed between data capture method (scanner) and data analysis method (software). All analyses were performed in SPSS version 25 (IBM Corporation, Armonk, NY, USA).

## Results

From the original data collected in the Netherlands, 76 surfaces were analyzed representing an average follow-up time of 36 months (SD 10.9). The data were initially analyzed using the previous gold-standard commercial software Geomagic. Laboratory profilometry data analyzed in Geomagic showed a median volume loss of –0.37 mm^3^ (IQR –3.75-2.30), whereas a median volume gain was observed for the intraoral scan data of +0.51 mm^3^ (IQR –2.17-4.26), representing a significant difference (*P*<.001). The median profile loss was 55.8 μm (IQR 24.43-77.60) and 43.65 μm (IQR 29.93-77.95) for the laboratory profilometer and intraoral scanner, respectively (*P*=.001). The maximum point loss on the occlusal surface was 398.4 μm (IQR 238.7-533.7) and 303.9 μm (IQR 217.6-483.0) for the profilometer and intraoral scan, respectively (*P*=.01).

Data from the freeware WearCompare showed a median volume change for the profilometer scan of –1.21 mm^3^ (IQR –3.48-0.56) and –0.39 mm^3^ (IQR –3.96-2.76) for the intraoral scan (*P*=.04). The median profile loss was 44.80 μm (IQR 29.48-91.63) and 43.10 μm (IQR 24.43-77.60) for the profilometer scan and intraoral scan, respectively (*P*=.18). The median maximum point loss on the occlusal surface for the profilometer scan was 317.1 μm (IQR 198.0-466.4) and was 278.3 μm (IQR 170.8-494.0) for the intraoral scan (*P*=.77). Therefore, no statistically significant differences were observed between the profilometer scans and intraoral scans when measurements were analyzed in WearCompare.

However, WearCompare detected significantly greater volume loss than analysis in Geomagic (*P*<.001), regardless of the scanner type. There were no differences between software in terms of average profile loss (*P*=.28) or maximum point loss (*P*=.26). When positive values were set to zero, the median volume change for the profilometer was unchanged, whereas intraoral scan volume loss evident. A significant difference was observed between the profilometry and intraoral scan data for Geomagic analysis (*P*=.02) but not for WearCompare analysis (*P*=.36) (Table 1).

**Table 1:** Volume changes (mm^3^) observed over 3 years when positive data were set to zero, which is a commonly used method by many commercial companies.

Scan type	Geomagic	WearCompare	*P* value
Laser profilometer, median (IQR)	–0.37 (–3.75-0.00)	–1.21 (–3.48-0.00)	.30
Intraoral scanner, median (IQR)	0.00 (–2.17-0.00)	–0.39 (–3.95-0.00)	.09

Moderate ICCs were observed in analyzing volume change data between the scanners and software types: 0.476 (95% CI 0.281-0.632) for Geomagic and 0.457 (95% CI 0.259-0.618) for WearCompare (both *P*<.001). WearCompare and Geomagic data showed slightly stronger ICC using the intraoral scanner (0.673, 95% CI 0.529-0.780; *P*<.001) than with the profilometer (0.525, 95% CI 0.341-0.671, *P*<.001).

## Discussion

### Principal Findings

This study demonstrates the differences in outcomes that can be observed when using low-resolution primary care digital scanners and precision measurements from hospital laboratory profilometers for measuring biological changes at the micron level. As expected, increased volume change values were observed using the higher-resolution and calibrated profilometer scans compared to the intraoral scans. Surprisingly, this difference was only statistically significant when using commercial software, previously considered to be the gold standard, for the analysis. The custom-built freeware outperformed the commercial software. The null hypothesis was therefore partially rejected. This finding suggests that if the analysis is conducted accurately, it may compensate for the decreased resolution of the scanner. This is a promising finding and has implications for the development of primary care systems.

There are several possible reasons for the reduced volume changes observed with intraoral scanners. Data interpolation or the mathematical averaging of data points across a surface can smoothen the topography of the surface and may overlook small discrepancies/areas of change in the surface. Smooth surface lesions, potentially on the buccal and lingual reference areas, will be subjected to heavy data undersampling internally in the scanner as the topography is not deemed to be as important. Smoothened surfaces are more susceptible to inaccuracies in data registration and alignment [15] as they will increase the mathematical tendency to minimize differences toward any sloped surfaces (in this case the occlusal surface). This can result in inaccuracies in alignment and biologically implausible outcomes. Analysis in a software that ignores features or the holistic geometric shape, such as Geomagic used in this study, will be particularly susceptible to this effect. Combining Geomagic analysis with the intraoral scan data resulted in an overall volumetric tooth tissue gain, which is physiologically impossible, indicating large errors within the analysis process.

For WearCompare, errors did not occur to the same extent, resulting in overall negative values for wear progression in both the profilometry and intraoral scans. Recent techniques developed at Radboud University involve using reference areas for alignment on the occlusal surfaces in addition to the buccal and lingual surfaces, providing additional control of the alignment along the Z axis. This may facilitate less translation and angular errors, and consequently less positive values. However, this comes at a tradeoff of increased analysis time and may also underestimate wear if an ICP algorithm is used for the Z axis. Further research will focus on validating this technique.

The correlation between wear measurements taken with the scanners was moderate as there are inherent but different errors for each form of data capture. Undetected, subvisual errors on casts or scans may have been present and subsequently analyzed as wear data. The profilometer is unable to scan undercuts, which means that less of the surface area can be used for selective surface alignment. In contrast, the intraoral scanner was successful at scanning undercuts. However, missing data or incomplete intraoral scans can also create errors whereby the triangle size is distorted and measurements can be skewed [25]. Recognizing where such errors may lie in each scan type will facilitate more accurate analysis.

Differences were observed between the profilometer and intraoral scan data when positive values were omitted from the analysis. Discounting positive values, which is commonly done with many types of commercial software and when profile loss and maximum point loss measurement metrics are reported, do not show error within the system. We observed that this can cause clinically significant changes to the outcome. Color maps of aligned scans can visually indicate areas of change, but the quantification does not always reflect the severity of progression. Discounting positive data increases the likelihood that a poor alignment will not be detected and wear underestimation or overestimation can occur. Reporting the negative changes may only be useful when trying to communicate wear to patients, but these metrics have limited diagnostic potential when measuring successive rates of wear.

### Limitations

This study has several limitations. The analysis was performed blinded to the sequence of scanning to limit bias. However, there were often indications of sequence, such as surface restorations or clear visual wear progression in the interim period. Although only one model of intraoral scanner was used, the hardware and software changed over the 3-year period of the study, emphasizing that research in this fast-moving field becomes rapidly outdated. Other intraoral scanners will have slightly different methods of processing missing data and interpolating irregularities, and it is possible that slightly different results may be achieved with different intraoral scanners. Single-tooth analysis was performed to maximize accuracy, which also limits generalizability to full-arch analysis. A large limitation in longitudinal wear analysis is that the true wear progression is unknown. One has to assume that wear has occurred and positive values represent errors in alignment or in the data capture process. This makes it difficult to identify any form of measurement as a gold standard.

### Conclusion

This study shows that low-resolution scanners can be used for measurements at the micron level provided appropriate analysis techniques and software are used. This could represent a step change in the way that erosive tooth wear is diagnosed and treated. From a dental point of view, the ability to view digital scans with increased magnification on a monitor also offers an increased diagnostic advantage. However, there is a duty of care on the profession and research community to not overestimate the quantitative capabilities of digital scanners to inform treatment or care outcomes until we are certain that they are adequately sensitive and specific to do so. This will depend on the level of accuracy required from the analysis process to diagnose disease progression within a feasible diagnostic window. The resolution and accuracy of primary care scanning tools is likely to increase rapidly and further work should concentrate on reducing the process errors inherent within each measurement system.

## Abbreviations

ICC: intraclass correlation
ICP: iterative closest point
